# The relationship of cervicothoracic mobility restrictions to fall risk and fear of falling in ankylosing spondylitis

**DOI:** 10.3389/fmed.2023.1159015

**Published:** 2023-06-27

**Authors:** Janine L. Johnston, Shane L. Harms, Glen T. D. Thomson

**Affiliations:** ^1^CIADS Research, Winnipeg, MB, Canada; ^2^Department of Ophthalmology and Medicine, Winnipeg, MB, Canada; ^3^Department of Family Medicine, University of Manitoba, Winnipeg, MB, Canada

**Keywords:** ankylosing spondylitis, biomechanics, cervical vertebrae, neck pain, range of motion

## Abstract

**Objective:**

The objective of this study is to determine whether restricted cervical mobility in ankylosing spondylitis (AS) is associated with increased fall frequency or fear of falling.

**Methods:**

A total of 134 AS patients and 199 age- and gender-matched control subjects (CS) with soft-tissue cervicothoracic pain were prospectively evaluated for fall risk. Subjects were divided into non-fallers, single fallers, and multiple fallers. Dynamic cervical rotations and static cervicothoracic axial measurements were compared between the groups. In total, 88 AS patients were reviewed more than once; Kaplan–Meier plots were constructed for fall risk as a function of cervical rotation amplitudes. Falls Efficacy Scale-International (FES-I) questionnaire measured the fear of falling.

**Results:**

In total, 34% of AS patients and 29% of CS fell (*p* = 0.271) in the year prior to evaluation. In AS, static anatomical measurements were unrelated to fall occurrence. The trends of multiple AS fallers to greater flexed forward postures and reduced dynamic cervical rotations were not statistically significant. Cervicothoracic pain (*p* = 0.0459), BASDAI (*p* = 0.002), and BASFI (*p* = 0.003) scores were greater in multiple fallers. FES-I scores were greater in fallers (*p* = 0.004). Of the 88 AS patients reviewed (or seen) on more than one occasion, 46.5% fell over the 9-year observation period, including all multiple fallers and 71.4% of single fallers. Survival curves showed increased fall risk as cervical rotational amplitudes decreased.

**Conclusion:**

In AS, decreased cervical rotations increase fall risk and fear of falling. In multiple fallers, falls were associated with greater disease activity. Cervical muscle stiffness in AS may cause non-veridical proprioceptive inputs and contribute to increased fall frequency similar to individuals with soft-tissue cervicothoracic pain.

## Introduction

Ankylosing spondylitis (AS) is a chronic inflammatory disease that primarily affects the axial skeleton, leading to vertebral deformities, progressive decreases in the range of spinal motion, and functional impairment of mobility. Increases in thoracic kyphosis are typical resulting from boney spinal changes in addition to soft tissue contractures (1) and increased cervical muscle tone (2), causing forward and downward displacement of the center of gravity and difficulty maintaining a line of sight at or above the horizon (3). As a result, falling is a major problem in patients with AS with an incidence of 13 to 25% (4–6) and a prevalence of 55% (7), leading to secondary morbidities and death (7, 8). Furthermore, the psychological fear of falling, even in individuals who have not actually fallen, can be almost as disabling as physical injuries which arise from falling (9). The relationship between static and dynamic balance and biomechanical changes in AS has been studied using various surrogate markers of fall risk (The Berg Balance Scale; balance platforms; timed mobility testing) rather than relating structural axial changes directly to the incidence of falls. Although the Bath Ankylosing Spondylitis Functional Index (BASFI) has been used in studies to assess physical functioning, it is self-reported and only contains one qualitative measure of cervical function (Question 8: Looking over your shoulder without turning your whole body) (10). Variability in AS patient populations and testing protocols has resulted in inconsistencies in outcomes, with some studies showing little or no association between biometric data and imbalance (1, 11, 12).

In the general population, many physical factors contribute to falling (9, 13, 14). Fall risk assessment includes a comprehensive neurological examination focusing on gait, lower limb strength, tone, sensation and coordination, vision, and cognition (13). Both acute and chronic neck pain cause imbalance through the disruption of cervical proprioceptive signals (15–17). Patients with chronic soft tissue pain and stiffness involving the neck, shoulders, and upper thorax are at increased risk for falls (18). Similar restrictions in the head and neck movements are seen in AS and are thought to result from cervical muscle hypertonicity occurring early in the course of the disease and underlying disease initiation and progression (2). Physiologically, chronic cervical immobility prolongs vestibulo-ocular reflex suppression during eye–head movements causing diminished gaze accuracy (19), potentially impairing navigation through complex visual environments where it is essential to accurately redirect gaze to novel targets. Practically, diminished neck rotation amplitudes double the risk of falling and contribute to increased fear of falling (18). Static axial deformities can also cause imbalance (20–22) but do not cause falls (18); rather thoracic kyphoscoliosis heightens fear of falling likely related to the impairment of trunk muscle co-activation needed to modulate gait-induced oscillations when walking (23).

We, therefore, sought to determine whether the chronically restricted cervical motion in AS is associated with increased fall frequency or fear of falling, similar to patients with chronically impaired cervical mobility due to soft tissue axial pain and stiffness. Additionally, we examined the contribution of abnormal cervicothoracic spinal morphology to fall causality and whether these static and dynamic axial abnormalities contribute differentially to falls or relate to disease duration and activity.

## Methods

Between June 2011 and March 2020, we examined 134 patients who attended a tertiary care rheumatology clinic for treatment of AS, with the approval of the CIADS Research Institutional Review Board in compliance with the Declaration of Helsinki and after obtaining informed written consent. All patients met the modified New York Criteria for AS (24). No patient had a previous or current history of vertigo, stroke, Parkinson's disease, ataxia, lower limb weakness, or cognitive impairment. Every patient completed the Falls Efficacy Scale-International (FES-I) 16-item balance questionnaire which provides a validated, quantitative measure of fear of falling (25). Those with a previous history of falling and those at greater fall risk have higher scores, indicating heightened fear of falling (low concern: 16–22/64; high concern: 23–64/64) (26).

A cohort of 199 patients presenting with soft tissue cervicothoracic musculoskeletal complaints but without structural spinal pathology served as control subjects (CS) and were age- and gender-matched to the patients with AS. A second cohort of AS fallers with a duration of disease since diagnosis of 15 years or less (*n* = 11) was matched to CS fallers with the same symptom durations.

Symptoms due to cervical mobility restriction and thoracic kyphoscoliosis were defined as chronic neck, shoulder, upper back, and forearm discomfort. Duration of disease for AS patients was based on when the patient was first diagnosed, whereas duration for CS was based on when the patient first recalled experiencing musculoskeletal pain or restriction of motion. Standardized systematic examination revealed any evidence of visual impairment, peripheral neuropathy, foot pain and lower limb osteoarthritis, or joint replacement. Drug history, including the number of medications or use of psychotropic medications, was obtained for each participant. Patients were segregated by fall frequency in the prior year, either no falls, one fall, or more than one fall (multiple falls). BASFI and BASDAI (Bath Ankylosing Spondylitis Disease Activity Index) were measured in the AS cohort assessing physical functioning and disability and patient perception of disease activity, respectively (10). Both BASFI and BASDAI are self-reported and use visual analog scales with values from 0 to 10 to denote increasing severity in symptoms or difficulty in task performance over the prior week. BASFI measures physical functioning and disability by assessing the ease of performance of standardized activities of daily living. BASDAI is a measure of disease activity assessing fatigue, neck, back, and hip pain, peripheral joint pain and swelling, and duration and severity of morning stiffness.

### Anatomical measurements

#### Static measurements

The static lateral inclination of the head and neck in roll (cervical tilt) was measured by a goniometer. Thoracic scoliosis was measured by scoliometer (Orthopedic Systems, Inc., Union City CA), with high scoliosis defined as cervicothoracic scoliosis with the apex of the curve rostral to T4; mid-thoracic scoliosis had an apex between T4 and T9, and low thoracic scoliosis had an apex between T9 and T12. Any scoliosis with at least two thoracic curves of opposite orientation was classified as complex. Kyphosis was an excessive posterior curvature of the thoracic spine and was either present or absent (27). In the AS cohort, tragus-to-wall distances (TWD) were also measured.

#### Kinetic measurements

All kinetic head and neck rotations were active and measured by a goniometer. Pitch movements around the interaural X-axis were measured from a vertical reference line between the vertex and sternal notch (Y-axis) (Supplementary Figure 1). Extension measurements were nose-up postures and were defined as negative if the region of head and neck motion was posterior to the Y-axis and positive if the head and neck did not extend back beyond the Y-axis. Flexion was a forward rotation of the head and neck. Total range of motion in pitch was calculated as the range of flexion minus the range of extension in degrees. Horizontal head and neck rotation was measured as left and right rotations in yaw around the Y-axis with total horizontal neck rotation as their sum. Total lateral head and neck flexion was roll rotation around the naso-occipital Z-axis, from the right ear down to the left ear down positions.

### Statistical analysis

Analysis of variance (ANOVA)/chi-square test for trend was used to examine for differences in continuous/categorical data between non-fallers, patients who had fallen once, and those who had fallen twice or more (multiple fallers). Student's *t*-test/Pearson's chi-square test was used to compare differences between continuous/categorical risk factors for non-fallers compared to all fallers (single plus multiple fallers). Odds ratios (ORs) for fall risk factors were calculated using binary logistic regression [Exp (B)], with any fall event as the outcome. Paired *t*-test/McNemar's test was used to compare continuous/categorical demographic and anatomical risk factors between the AS cohort and their paired control subjects.

Hierarchical multiple regression assessed the ability of anatomical factors to predict FES-I scores in fallers. Least Absolute Shrinkage and Selection Operator (Forward criterion: the probability of F to enter ≤ 0.05) was used to determine which of the other variables known to contribute to increased fall risk was relevant; these included age, impaired vision, number of psychotropic medications, the total number of medications, lower limb osteoarthritis and joint replacement, peripheral neuropathy, foot pain, fatigue, and other comorbidities. Of these, age and medication number were the only significant independent contributors.

Partial correlation assessed any relationship between the duration of symptoms, kinetic and static axial measurements, BASDAI/BASFI scores, and FES-I scores, controlling for age.

A total of 88 AS patients were reviewed on two or more occasions over the duration of the study: 59 non-fallers, 21 single fallers, and 8 multiple fallers. Kaplan–Meier plots of the number of patients at risk of falling vs. the amplitude of total head and neck sagittal and horizontal rotations in degrees were calculated.

For all statistical analyses, a two-sided *p*-value of ≤ 0.05 was considered significant. Statistical analysis was performed with IBM SPSS, version 28. SQUIRE reporting guidelines were used in the preparation of this manuscript (28).

## Results

### Subject characteristics: ankylosing spondylitis patients

Of the 134 AS patients, 88 did not suffer any falls over the year prior to assessment (Table 1). In total, 31 patients (23%) fell once and 15 patients (11%) suffered multiple falls. There was no significant difference between mean ages for non-fallers compared to fallers, and age did not confer an additional risk of falling (OR 0.99; 95% CI 0.96–1.01; *p* = 0.281). There was a male predominance of 1.9:1 in the group as a whole. No gender effect was detected with respect to fall risk (OR 1.51; 95% CI 0.72–3.16; *p* = 0.276).

**Table 1:** Ankylosing spondylitis subject characteristics.

	**Total**	**Non-faller group**	**Single faller group**	**Multiple faller group**	**Overall p**	**All fallers**	**Overall p**
	**(n = 134)**	**(n = 88)**	**(n = 31)**	**(n = 15)**		**(n = 46)**	
Age, mean ± SD (years) (range)	47.9 ± 14.4 (16–80)	48.9 ± 15.1	45.5 ± 13.3	47.1 ± 12.6	0.22^‡^	46.0 ± 12.9	0.28^*^
Women, no. (%)	47 (35.1%)	28 (31.8%)	13 (41.9%)	6 (40.0%)	0.34^†^	19 (41.3%)	0.28^†^
Symptomatic Cervical/Thoracic Pain/Stiffness, no. (%)	73 (54.9%)	46 (52.3%)	15 (48.4%)	12 (80.0%)	0.13^†^	27 (58.7%)	0.40^*^
Duration since diagnosis, mean ± SD (years)	26.3 ± 14.6	26.8 ± 15.1	24.8 ± 13.8	26.9 ± 13.8	0.79^‡^	25.5 ± 13.7	0.63^*^
FES-I score, mean ± SD (range 16 to 64)	21.8 ± 8.5	20.0 ± 5.9	22.4 ± 9.7	31.0 ± 12.0	< 0.0001^‡^	25.2 ± 11.1	0.004^*^
BASDAI (range 0–10)	4.5 ± 2.2 (0.2–9.1)	4.3 ± 2.1 (0.2–8.7)	4.2 ± 2.1 (1.0–7.1)	6.5 ± 2.2 (1.7–9.1)	0.002^‡^	5.0 ± 2.4 (1.0–9.1)	0.12^*^
BASFI (range 0–10)	3.4 ± 2.5 (0–9.3)	3.2 ± 2.4 (0–8.8)	2.9 ± 2.3 (0.3–8.2)	5.6 ± 2.6 (0.7–9.3)	0.002^‡^	3.8 ± 2.7 (0.3–9.3)	0.29^*^

^*^Student's t-test. ^†^Chi square/Chi square for trend. ^‡^ANOVA.

The occurrence of symptoms of neck and upper back pain and stiffness was not significantly greater for fallers compared to their non-falling counterparts. However, a greater proportion (80%) of multiple fallers had symptomatic cervicothoracic pain compared to non-fallers (52.3%) (*p* = 0.045). Fallers did not have longer mean symptom duration since diagnosis than non-fallers.

FES-I questionnaire scores were significantly greater in fallers compared to non-fallers. Scores were also significantly greater for multiple fallers compared to non-fallers (*p* < 0.0001 Tukey's *post hoc* test) and single fallers (*p* = 0.002 Tukey's *post hoc* test). Fallers showed high concern about the possibility of falling (FES-I score >23) compared to non-fallers.

Neither BASDAI nor BASFI measurements were greater in fallers compared to non-fallers, but multiple fallers had significantly greater BASDAI scores than non-fallers (*p* = 0.002 Tukey's *post hoc* test) and single fallers (*p* = 0.004 Tukey's *post hoc* test). Multiple fallers also had significantly greater BASFI scores than non-fallers (*p* = 0.003, Tukey's *post hoc* test) and single fallers (*p* = 0.002, Tukey's *post hoc* test).

Among other fall risk factors, multiple or psychotropic drug use, other comorbidities, visual impairment, foot pain, lower limb osteoarthritis, peripheral neuropathy, and joint replacements were not significantly greater in fallers compared to their non-falling counterparts.

### Anatomical risk factors

#### Falling

No static anatomical measurement showed any relationship with fall occurrence (Table 2). All patients had stationary flexed-forward resting postures of the head and neck that did not vary between the groups. While multiple fallers tended to have greater static flexed-forward posture, TWD measurements, and increased frequency of kyphosis, these did not reach statistical significance. Similarly, multiple fallers also had increased frequency of complex scoliosis, slightly larger high and low thoracic scoliosis mean amplitudes, and reduced dynamic head and neck movement amplitudes compared to non-fallers and single fallers, again not achieving statistical significance.

**Table 2:** Anatomical factors segregated by fall frequency.

**Anatomical risk factors**							
**Mean** ±**SD**	**Total**	**Non-faller group**	**Single faller group**	**Multiple faller group**	**Overall p**	**All fallers**	**Overall p**
	**(*****n** =* **134)**	**(*****n** =* **88)**	**(*****n** =* **31)**	**(*****n** =* **15)**		**(*****n** =* **46)**	
**Static measurements**							
Cervical Tilt (deg)	0.5 ± 1.8	0.7 ± 1.8	0.3 ± 2.0	0 ± 1.7	0.26^‡^	1.7 ± 1.8	0.12^*^
Neck Resting Posture (deg)	39.9 ± 8.7	40.0 ± 8.6	38.6 ± 8.1	42.1 ± 10.2	0.43^‡^	39.8 ± 8.9	0.86^*^
Scoliosis (deg)							
High thoracic scoliosis	2.8 ± 2.3	2.6 ± 2.2	2.8 ± 2.6	3.7 ± 2.2	0.24^‡^	3.1 ± 2.5	0.30^*^
Mid thoracic scoliosis	2.3 ± 2.5	2.4 ± 2.6	2.2 ± 2.4	1.6 ± 2.6	0.53^‡^	2.1 ± 2.4	0.43^*^
Low thoracic scoliosis	1.9 ± 2.4	1.9 ± 2.5	1.5 ± 2.1	2.6 ± 2.4	0.37^‡^	1.9 ± 2.3	0.99^*^
Any Thoracic Scoliosis ≥ 5 deg, no (%)	80 (59.7%)	54 (61.4%)	16 (51.6%)	10 (66.7%)	0.92^†^	26 (56.5%)	0.59^†^
Complex Scoliosis, no (%)	63 (47.0%)	42 (47.7%)	11 (52.4%)	10 (66.7%)	0.56^†^	21 (45.7%)	0.82^†^
Kyphosis, no. (%)	103 (76.9%)	68 (77.3%)	22 (71.0%)	13 (86.7%)	0.74^†^	35 (76.1%)	0.88^†^
Tragus to Wall distance (cm)	15.0 ± 5.4	15.3 ± 5.7	14.5 ± 5.6	16.6 ± 6.7	0.48^‡^	15.2 ± 6.0	0.78^*^
**Dynamic measurements**							
Neck Movements (deg)							
Neck extension	14.6 ± 20.2	15.3 ± 20.7	10.1 ± 18.6	20.0 ± 19.9	0.43^‡^	13.3 ± 19.4	0.60^*^
Neck flexion	71.3 ± 13.1	71.4 ± 12.0	71.7 ± 17.5	69.8 ± 9.4	0.89^‡^	71.1 ± 15.3	0.92^*^
Full neck pitch	56.7 ± 27.8	56.8 ± 27.6	60.2 ± 29.9	48.3 ± 24.6	0.40^‡^	56.3 ± 28.6	0.92^*^
Horizontal neck rotation left	46.0 ± 17.8	45.9 ± 18.2	47.9 ± 18.5	42.5 ± 13.8	0.62^‡^	46.2 ± 17.1	0.95^*^
Horizontal neck rotation right	33.8 ± 13.5	33.8 ± 13.6	34.2 ± 14.8	32.7 ± 9.7	0.94^‡^	33.7 ± 13.2	0.95^*^
Full horizontal neck rotation	80.2 ± 30.9	80.6 ± 31.0	80.8 ± 34.7	76.8 ± 22.1	0.90^‡^	79.5 ± 31.0	0.85^*^
Full lateral flexion	44.0 ± 19.5	45.4 ± 18.8	42.4 ± 22.6	39.1 ± 16.3	0.45^‡^	41.4 ± 20.6	0.27^*^

^*^Student's t-test; ^†^Chi square/Chi square for trend; ^‡^ANOVA.

#### FES-I

When assessing the contribution of various risk factors to the variance in FES-I scores among fallers, age and medication number significantly and independently contributed to fall risk, explaining 33.0% of the total 41.4% variance in FES-I scores [F_(2, 43)_ = 15.188, *p* < 0.0001]. Neck flexion was the only significant anatomical risk factor that contributed to 8.4% of the variance in FES-I scores in fallers (beta = −0.30).

There was a significant positive correlation between FES-I scores and BASDAI scores for both non-fallers (*r* = 0.423; *p* = 0.0001) and fallers (*r* = 0.485; *p* = 0.001). There was a similar positive correlation between FES-I scores and BASFI scores for both non-fallers (*r* = 0.504; *p* < 0.0001) and fallers (*r* = 0.693; *p* < 0.0001).

#### Symptom duration

For non-fallers, there were no significant correlations between any static or dynamic anatomical measurements and duration of symptoms controlling for age. For fallers as a group, the duration of symptoms did not correlate with any dynamic neck movements. Low-thoracic scoliosis amplitudes correlated positively with duration for all fallers (*r* = 0.324; *p* = 0.030). There was a positive correlation between symptom duration and BASDAI scores (*r* = 0.871; *p* = 0.0001) and BASFI scores (*r* = 0.754; *p* = 0.003) for multiple fallers only, after controlling for age. Duration of symptoms did not correlate with FES-I scores for non-fallers or fallers, including multiple fallers.

#### Repeated measurements

**Figure 1:**
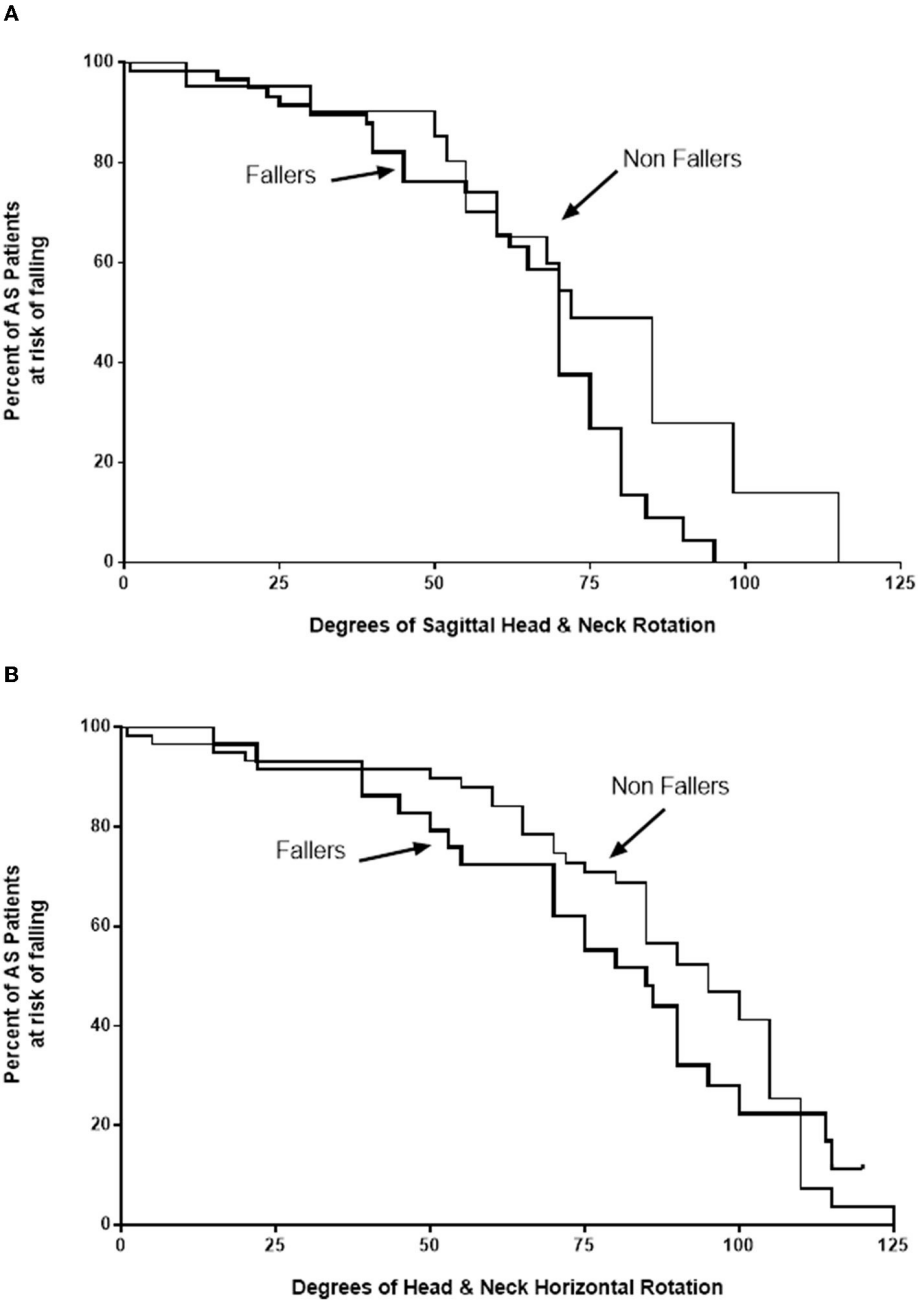
Kaplan–Meier survival curves. Kaplan–Meier survival curves show the risk of falling for both non-fallers with ankylosing spondylitis (AS) (thin lines) and those who had previously fallen (thick lines) increases as both head and neck sagittal (pitch) amplitudes **(A)** and horizontal rotation amplitudes **(B)** diminish. The curves are significantly different between non-fallers and fallers for both pitch (*p* = 0.0463) and horizontal rotation (*p* = 0.0012). As rotational amplitudes diminish, the risk of falling is higher for non-fallers compared to fallers, with Mantel–Haenszel hazard ratios of 2.417 for sagittal rotation and 3.438 for horizontal rotation.

Of the 88 AS patients who were seen on more than one occasion, 59 had not fallen at the time of initial evaluation; and 29 had fallen, 21 once and 8 two or more times (Figure 1). Of the non-faller group, 41 never fell over the 9-year observation period and 18 (30.5%) fell one or more times. Only 6 of the 21 initial single fallers did not fall again over the observation period. Of the 8 initial multiple fallers, all 8 fell again, 5 fell once, and 3 fell twice or more. Kaplan–Meier survival curves showed that the risk of falling for both non-fallers and fallers increased as both head and neck pitch (Figure 1A) and yaw (Figure 1B) amplitudes decreased. The curves for non-fallers are significantly different from fallers for both sagittal (*p* = 0.0463) and horizontal rotation (*p* = 0.0012). As rotational amplitudes decrease, there is a greater risk of falling for non-fallers compared to fallers for both sagittal rotations (Mantel–Haenszel Hazard ratio 2.417; 95% CI 1.015–5.756) and horizontal rotations (Mantel–Haenszel Hazard ratio 3.438; 95% CI 1.629–7.256).

#### Paired cohort

There were no significant differences between AS patients and their matched control cohort with soft tissue rheumatic complaints with respect to gender and age (Table 3). The proportion of patients who were symptomatic from their disease process was similar for both groups as a whole and for non-fallers. However, among fallers, there were a significantly greater number of symptomatic individuals in the matched CS than in the AS cohort. The mean duration of symptoms (since diagnosis) was significantly longer for AS patients compared to CS, even though the groups were age-matched at the time of evaluation. This occurred because AS patients were younger at the age of symptom onset.

**Table 3:** Matched ankylosing spondylitis and control patients demographic and biometric measurements.

**Mean ±SD**	**AS total**	**Control total**	***p***	**AS non-faller group**	**Control non-faller group**	**p**	**AS faller group**	**Control faller group**	***p***
	**n** = **134**	**n** = **199**		**n** = **88**	**n** = **142**		**n** = **46**	**n** = **57**	
Age, mean ± SD (years)	50.4 ± 14.7	50.5 ± 14.8	0.95^*^	48.9 ± 15.1	50.7 ± 14.1	0.36^*^	46.0 ± 12.9	50.0 ± 16.5	0.18^*^
Women, no. (%)	47 (35.1%)	93 (45.6%)	1.000^†^	28 (31.8%)	68 (47.9%)	0.02^†^	19 (41.3%)	25 (44.6%)	0.79^†^
Symptomatic cervical/thoracic pain/stiffness, no. (%)	73 (54.9)%	127 (62.3%)	0.09^†^	46 (52.3%)	83 (58.5%)	0.36^†^	24 (58.7%)	44 (78.6%)	0.04^†^
Age of symptom onset ± SD (years)	21.8 ± 7.4	40.9 ± 15.8	< 0.0001^*^	22.5 ± 8.4	41.8 ± 15.4	< 0.0001^*^	20.4 ± 5.8	38.5 ± 16.5	< 0.0001^*^
Duration, mean ± SD (years)	28.3 ± 14.6	9.6 ± 10.4	< 0.0001^*^	26.8± 15.1	8.2 ± 8.5	< 0.0001^*^	25.5 ± 13.7	13.0 ± 13.7	< 0.0001^*^
FES-I score, mean ± SD (range 16-64)	22.6 ± 9.1	20.7 ± 7.4	0.03^*^	20.0 ± 5.9	19.6 ± 6.4	0.64^*^	25.2 ± 11.1	23.5 ± 8.9	0.50^*^
Cervical tilt (deg)	0.4 ± 1.8	1.2 ± 1.9	< 0.0001^*^	0.7 ± 1.7	1.2 ± 1.9	0.04^*^	0.2 ± 1.8	1.3 ± 1.9	0.004^*^
Neck resting posture (deg)	40.3 ± 9.0	37.2 ± 5.3	< 0.0001^*^	40.0 ± 8.6	36.9 ± 5.3	0.001^*^	39.8 ± 8.9	38.1 ± 5.1	0.23^*^
Scoliosis (deg)									
High thoracic scoliosis	2.8 ± 2.3	2.6 ± 2.4	0.52^*^	2.6 ± 2.2	2.6 ± 2.3	1.000^*^	3.1 ± 2.5	2.6 ± 2.5	0.32^*^
Mid thoracic scoliosis	2.1 ± 2.5	2.5 ± 2.8	0.20^*^	2.4 ± 2.5	2.4 ± 2.6	1.000^*^	2.0 ± 2.3	2.6 ± 3.1	0.37^*^
Low thoracic scoliosis	1.9 ± 2.4	1.8 ± 2.4	0.58^*^	1.9 ± 2.5	1.7 ± 2.4	0.55^*^	1.9 ± 2.3	1.9 ± 2.4	1.000^*^
Any thoracic scoliosis ≥ 5 deg, no (%)	117 (56.3%)	127 (64.1%)	0.09^†^	54 (61.4%)	91 (64.1%)	0.33^†^	26 (56.5%)	36 (64.3%)	0.27^†^
Complex scoliosis, no (%)	98 (47.1%)	104 (52.5%)	0.43^†^	42 (47.7%)	76 (53.5%)	0.36^†^	21 (45.7%)	30 (51.7%)	0.62^†^
Kyphosis, no. (%)	164 (78.8%)	158 (79.8%)	1.000^†^	68 (77.3%)	111 (78.2%)	0.43^†^	35 (76.1%)	48 (82.8%)	0.69^†^
Neck movements (deg)									
Neck extension	15.7 ± 20.4	5.4 ± 10.6	< 0.0001^*^	15.3 ± 20.7	5.6 ± 9.8	< 0.0001^*^	13.3 ± 19.4	4.9 ± 12.5	0.01^*^
Neck flexion	70.8 ± 13.2	78.8 ± 9.1	< 0.0001^*^	71.4 ± 12.0	79.7 ± 8.8	< 0.0001^*^	71.1 ± 15.3	76.9 ± 9.5	0.02^*^
Full neck pitch	54.7 ± 27.8	73.7 ± 15.0	< 0.0001^*^	55.8 ± 27.6	74.4 ± 13.9	< 0.0001^*^	55.3 ± 28.6	71.8 ± 17.4	0.001^*^
Horizontal neck rotation left	44.1 ± 18.6	53.8 ± 10.5	< 0.0001^*^	45.9 ± 18.2	53.8 ± 10.1	< 0.0001^*^	46.2 ± 17.1	53.8 ± 11.5	0.01^*^
Horizontal neck rotation right	32.7 ± 14.1	38.2 ± 8.3	< 0.0001^*^	33.8 ± 13.6	37.9 ± 8.0	0.004^*^	33.7 ± 13.2	39.1 ± 9.0	0.02^*^
Full horizontal neck rotation	77.0 ± 32.5	92.0 ± 17.0	< 0.0001^*^	80.8 ± 30.9	91.6 ± 16.7	0.001^*^	79.5 ± 31.0	93.0 ± 17.8	0.007^*^
Full lateral flexion	42.2 ± 20.0	48.1 ± 16.8	< 0.0001^*^	45.4 ± 18.8	47.8 ± 16.5	0.31^*^	41.4 ± 20.6	48.9 ± 17.6	0.05^*^

^*^Paired T-Test/Student T-Test. ^†^McNemar Test/Pearson Chi-Square.

FES-I questionnaire scores were significantly higher in the AS group. Even though FES-I scores for fallers showed high concern about falling (>23), there was no any difference in the number of AS patients who actually fell (34%) compared to the CS group (29%) (*p* = 0.271) nor there was any difference in the numbers of multiple fallers between the groups (*p* = 0.374).

For static measurements, forward flexion of the head at rest was greater for the AS group as a whole but was not greater for fallers. Cervical tilt was significantly more impaired in the AS group. There were no differences in any thoracic scoliosis amplitudes, frequency of kyphosis, complex curves, or any thoracic scoliosis > 5 degrees between the groups.

All dynamic head movements were significantly more restricted in AS patients than in CS, likely due to the more significantly prolonged duration of the disease for the AS group. Accordingly, AS fallers with durations of 15 years or less (6.6 years, SD 4.3, range 1–14) were matched with CS fallers of similar durations (6.7 years, SD 4.1, range 1–14). There were only 11 single and multiple AS fallers who met this criterion. Between these groups, all dynamic head rotations including horizontal rotations (*p* = 0.486), lateral flexion (*p* = 0.523), and sagittal rotations (*p* = 0.994) were not significantly different.

## Discussion

A number of studies have described increased imbalance in AS patients compared to healthy control subjects without arthritic disorders. Using standardized measures of balance and dynamic mobility (5, 6, 29, 30) or assisted balance testing devices (31–37), these studies have shown impairment of both static and dynamic balance and have correlated these measures of imbalance with deficits in the sagittal alignment, typically thoracic kyphosis. Other studies have shown no strong association between biometric data and imbalance (1, 11, 12). None have correlated imbalance or biometric data with falling; that is, AS patients appeared to be at increased fall risk, but it was not determined as to whether this manifested as actual falls. Therefore, while we did not specifically measure balance by any standardized measure, we sought to determine whether AS patients actually do fall and if so, whether is there a relationship between fall frequency and restrictions in cervicothoracic mobility. Our results confirmed that diminished dynamic cervical movements, not abnormal static cervicothoracic axial restrictions, are associated with both increased falling and increased fear of falling in support of those studies which have shown an association between abnormal biometrics and imbalance in AS patients.

While Çinar et al. (29) suggested that poor balance was not common in AS patients, Martindale et al. (7) showed that greater than 50% of AS patients experienced falls after being diagnosed. Our study showed an overall fall incidence of 34% in the AS cohort with no apparent effect of age, gender, or duration of the disease since diagnosis. However, falling in our AS cohort did not differ from the CS group with soft tissue restrictions of cervical mobility. Alkan et al. (4) noted a history of falls in 20% of their AS cohort compared to 5% in age- and gender-matched healthy control groups, indicating that both AS patients and matched CS with soft tissue restrictions of cervical mobility had much higher fall frequencies than healthy individuals.

To date, only four studies have specifically measured balance and cervical rotation in AS as part of a group of spinal mobility measurements with variable results (11, 30–32). Vergara et al. (32) showed that standing displacement of the center of pressure in the sagittal plane was negatively related to cervical horizontal rotation and positively related to forward flexion. Unver et al. (30) found a weak positive correlation with measures of static and dynamic balance and cervical rotation but did not find any association between these measures of balance and flexed forward posture. Conversely, Sawacha et al. (31) showed diminished cervical flexion, extension, and lateral inclination compared to control subjects, but horizontal cervical rotation was not reduced. This study did not correlate cervical rotation amplitudes with their posturographic measures of balance. Finally, Aydog et al. (11) did not find any correlation between cervical rotation and measures of dynamic or postural stability tested with a moveable balance platform. We expected to see a similar relationship between fall frequency and cervical immobility in AS patients as in subjects with soft tissue contractures alone, but there were no differences in any cervical rotational amplitudes between AS patients who fell and those who did not, likely because of the prolonged disease durations of all AS patients. However, although statistical significance is not achieved, multiple fallers had increased dynamic restrictions in all three planes of cervical motion compared to non-fallers. They also had a trend toward increased flexed forward posture, increased incidence of kyphosis, and increased TWD, similar to previous studies showing imbalance in AS patients with increased thoracic kyphosis (31, 34–36).

Although AS patients did not fall any more frequently than CS, fear of falling among AS patients was greater, indicating significant concern about the possibility of falling again, similar to the study by Dursun et al. (5). Despite increased concern about falling, if an AS patient has fallen, the likelihood is that they will fall again. Over time, as cervical rotational amplitudes decreased in both yaw and pitch, fall risk in our cohort of AS patients increased. Interestingly, the risk of falling was approximately 2–3 times greater in non-fallers than in fallers. That is, those who had not fallen when assessed initially were more likely to fall compared to those who had already fallen prior to their enrolment in the study. While it is important to assure that AS patients who have fallen do not fall again, similar care should be taken to protect AS patients who have not fallen, as with progressively diminishing cervical rotation amplitudes, they are at significant fall risk.

While subjective measures of disease activity and functional deficits, including symptomatic cervical and thoracic pain and stiffness, BASDAI and BASFI scores were not greater in AS fallers compared to non-fallers, these measures were significantly worse in multiple fallers compared to both non-fallers and single fallers. Similarly, FES-I scores correlated positively with both BASDAI and BASFI scores, indicating that fear of falling related to both subjective disease activity and physical functioning. There are significant differences between previous studies which have shown a limited (37) or no relationship between disease activity and measures of imbalance (1, 34, 36), and studies that have shown positive correlations between BASDAI and BASFI and fall risk or fall frequency (4–6). This may relate to the fact that most studies did not actually determine the frequency of falling in their AS cohorts, and patients who fell were not evaluated separately from their non-falling counterparts. Only one study (5) has shown AS fallers to have higher Bath Ankylosing Spondylitis Metrology Index (BASMI) scores than non-fallers, and the BASMI score to correlate positively with the number of falls. BASMI measurements include tragus-to-wall distances and cervical rotation amplitudes and are used to define significant clinical changes in spinal mobility (10).

The neck proprioceptive system is designed to stabilize head-on-trunk movement, unlike the vestibular system which is designed to stabilize gaze. A mismatch between smaller vestibular signals due to increased neck muscle viscosity and larger non-veridical proprioceptive signals due to cervical muscle co-activation causes changes in eye–head kinematics, leading to increased fall risk (19). Aydog et al. (11) speculated that enthesopathy in AS may damage afferent nerve fibers in ligaments, tendons, and joint capsules, thereby disturbing proprioception. Conversely, Swinkels and Dolan (38) did not find any loss of thoracolumbar or sacral proprioception with increasing disease activity and decreasing spinal mobility. Cervical muscle spindles, especially those of deep cervical muscles, are the major proprioceptive organs of the neck (39). A recent analysis of muscle mechanical properties in patients with AS has shown increased stiffness and tone and decreased relaxation of cervical muscles compared to control subjects; muscle stiffness was inversely correlated with cervical rotation and directly correlated with BASDAI (2). This hypertonicity occurs early in the course of the disease and may underlie disease initiation and progression. Our cohort of AS patients had similar static cervical restrictions and fall frequency to CS with soft-tissue restrictions of cervical motion; dynamic cervical rotation amplitudes were not different between the groups of fallers controlling for disease duration. Additionally, multiple AS fallers trended toward having greater static flexed-forward posture and reduced dynamic head and neck movement compared to non-fallers. Finally, greater restrictions in cervical rotations caused greater fall risk in AS patients over time. We might, therefore, speculate that the same problem with non-veridical proprioceptive inputs from hypertonic, stiff cervical muscles may be occurring in AS patients as it occurs in patients with chronic soft tissue restrictions of cervical muscles. That is, cervical muscle contracture in AS is the source of inaccurate, not deficient proprioceptive inputs and vestibular mismatch, and is an underlying cause of imbalance and falls.

While this study small and recall bias resulting from errors in recollection of patient falls within the prior year may be limitations, the relationship between falling, risk of falling, and decreasing cervical rotation amplitudes in AS is similar to fall risk relationships in patients with cervical muscle restrictions but without AS pathology.

## Conclusion

Our study has confirmed our primary objective: In patients with AS, diminished dynamic movements of the head and neck in the horizontal and sagittal planes are associated with both increased falling and increased fear of falling. Static cervicothoracic axial measurements do not contribute to increased fall frequency or fear of falling. Most AS patients who have fallen at least once will fall again and over time, those who have not fallen have a two- to three-fold greater risk of falling with decreasing cervical rotational amplitudes. Fall risk is associated with disease activity but not disease duration. These restrictions in dynamic cervical rotation and the associated risk of falling are similar to those seen in individuals with soft tissue pain and stiffness and likely have the same pathophysiology with respect to non-veridical proprioceptive input from cervical muscles that are stiff and hypertonic as a result of the primary disease process. Physical treatment should be aimed at increasing neck mobility, reducing tone and stiffness, and improving muscle elasticity in order to reduce fall risk.

## Data availability statement

The original contributions presented in the study are included in the article/Supplementary material, further inquiries can be directed to the corresponding author.

## Ethics statement

The studies involving human participants were reviewed and approved by CIADS Research Ethics Committee #0012011. The patients/participants provided their written informed consent to participate in this study.

## Author contributions

JJ and GT conceived the study. GT examined the patients and undertook the measurements. GT, JJ, and SH carried out the statistics and wrote and edited the manuscript. All authors contributed to the article and approved the submitted version.

## Supplementary material

Supplementary Figure 1 Measurements of head and neck rotations. Pitch: Measurements for head and neck movements in pitch were made from a vertical reference line between the vertex and the sternal notch (circle). Extension measurements were negative if the active range of motion was posterior to the vertical reference line. Flexion was a forward rotation of the head and neck from the vertical reference line. The total active range of motion in pitch was the amplitude of flexion minus the amplitude of extension in degrees. Roll: Fulhead and neck lateral flexion was total active rotation around the naso-occipital Z axis in roll, from the maximum **right ear** down position to the maximum **left ear** down position in degrees. Yaw: Horizontal neck rotation was measured as maximal active **left** and **right** rotations in degrees around the Y axis with total horizontal neck rotation as their sum.
