# Supplementary figures and images for: The relationship of cervicothoracic mobility restrictions to fall risk and fear of falling in ankylosing spondylitis
Source: Front Med (Lausanne). 2023 Jun 27;10:1159015. doi: 10.3389/fmed.2023.1159015 (PMC10333576; doi:10.3389/fmed.2023.1159015)

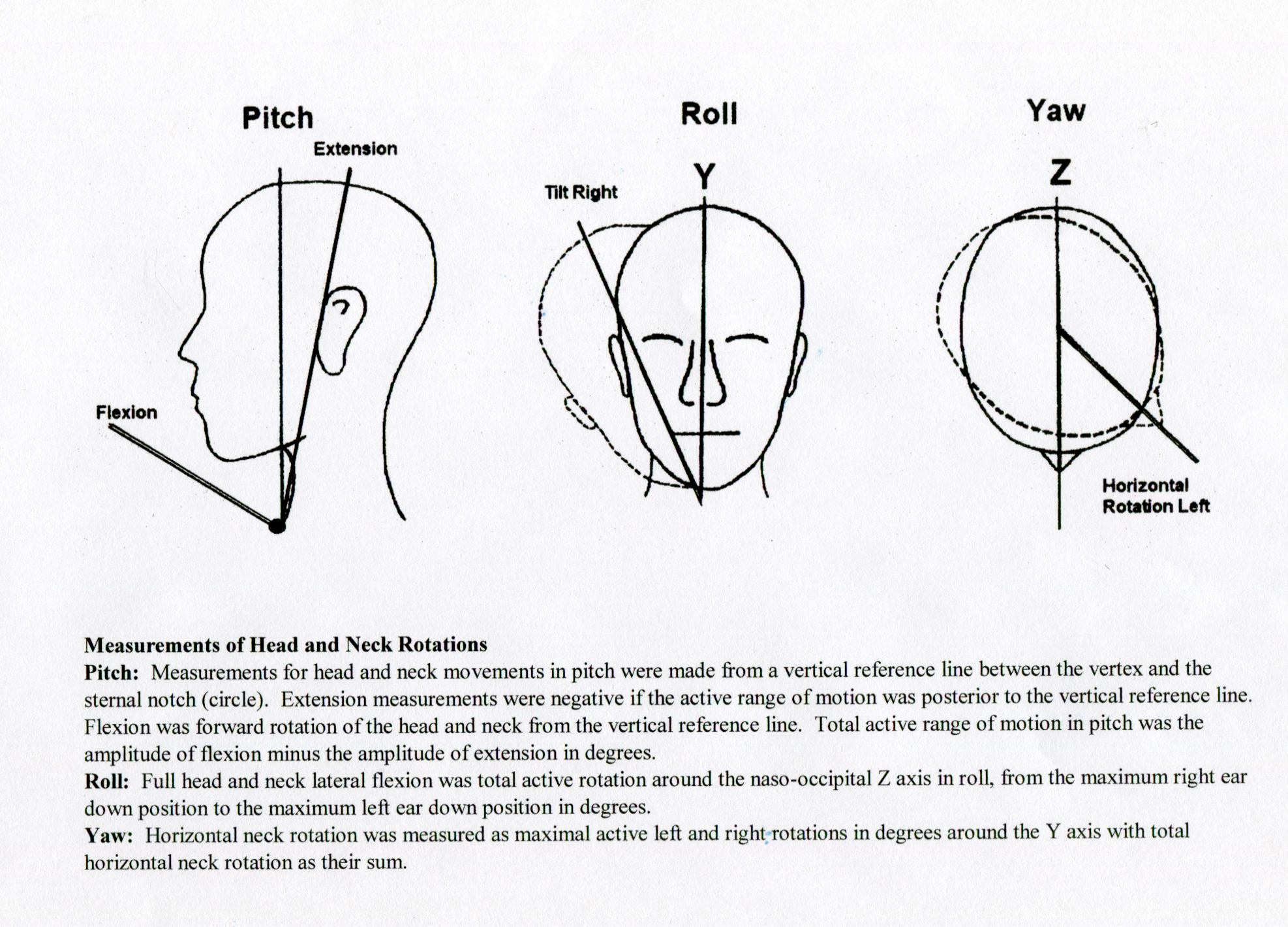

Supplement: Supplementary Figure 1 — Measurements of head and neck rotations. Pitch: Measurements for head and neck movements in pitch were made from a vertical reference line between the vertex and the sternal notch (circle). Extension measurements were negative if the active range of motion was posterior to the vertical reference line. Flexion was a forward rotation of the head and neck from the vertical reference line. The total active range of motion in pitch was the amplitude of flexion minus the amplitude of extension in degrees. Roll: Fulhead and neck lateral flexion was total active rotation around the naso-occipital Z axis in roll, from the maximum right ear down position to the maximum left ear down position in degrees. Yaw: Horizontal neck rotation was measured as maximal active left and right rotations in degrees around the Y axis with total horizontal neck rotation as their sum. [file Image_1.tif]
